# A molecular signature of lung-resident CD8^+^ T cells elicited by subunit vaccination

**DOI:** 10.1038/s41598-022-21620-7

**Published:** 2022-11-09

**Authors:** Naveenchandra Suryadevara, Amrendra Kumar, Xiang Ye, Meredith Rogers, John V. Williams, John T. Wilson, John Karijolich, Sebastian Joyce

**Affiliations:** 1 Department of Veterans Affairs, Tennessee Valley Healthcare Center, Nashville, TN 37212 USA; 2 Department of Pathology, Microbiology and Immunology, Vanderbilt University Medical Center, Nashville, TN 37232 USA; 3 Department of Chemical and Biomolecular Engineering and Department of Biomedical Engineering, Vanderbilt University, Nashville, TN 37212 USA; 4 Department of Paediatrics, University of Pittsburgh School of Medicine, Pittsburgh, PA 15224 USA; 5 Institute for Infection, Immunity, and Inflammation in Children (i4Kids), Pittsburgh, PA 15224 USA

**Keywords:** Immunology, Adaptive immunity, Antimicrobial responses, Vaccines

## Abstract

Natural infection as well as vaccination with live or attenuated viruses elicit tissue resident, CD8^+^ memory T cell (Trm) response. Trm cells so elicited act quickly upon reencounter with the priming agent to protect the host. These Trm cells express a unique molecular signature driven by the master regulators—Runx3 and Hobit. We previously reported that intranasal instillation of a subunit vaccine in a prime boost vaccination regimen installed quick-acting, CD8^+^ Trm cells in the lungs that protected against lethal vaccinia virus challenge. It remains unexplored whether CD8^+^ Trm responses so elicited are driven by a similar molecular signature as those elicited by microbes in a real infection or by live, attenuated pathogens in conventional vaccination. We found that distinct molecular signatures distinguished subunit vaccine-elicited lung interstitial CD8^+^ Trm cells from subunit vaccine-elicited CD8^+^ effector memory and splenic memory T cells. Nonetheless, the transcriptome signature of subunit vaccine elicited CD8^+^ Trm resembled those elicited by virus infection or vaccination. Clues to the basis of tissue residence and function of vaccine specific CD8^+^ Trm cells were found in transcripts that code for chemokines and chemokine receptors, purinergic receptors, and adhesins when compared to CD8^+^ effector and splenic memory T cells. Our findings inform the utility of protein-based subunit vaccination for installing CD8^+^ Trm cells in the lungs to protect against respiratory infectious diseases that plague humankind.

## Introduction

Memories of past encounters with pathogens is a key feature of the adaptive immune system. As memory cells act quickly to contain infection, this feature is leveraged in successful vaccination strategies. Memory CD8^+^ T cells are essential for the control and clearance of many pathogens that have an intracellular lifecycle—e.g., all viruses, certain bacteria—such as *Francisella, Listeria*, *Mycobacteria,* and others, as well as parasites—such as *Plasmodium, Toxoplasma*, et cetera. Infection begins with a breach of barrier tissues, such as the skin and the mucosae of the lungs, intestine and colon, and reproductive tract, and the entry of pathogens inside cells. Thus, these vulnerable barriers require constant immune surveillance to maintain tissue homeostasis^1–15^ (see also ref.^16^). Immune surveillance begins with the capture of pathogens by tissue-resident antigen-presenting cells, which, upon activation, migrate to the local lymphoid organ, and process and present antigen to activate rare antigen-specific naïve precursor CD8^+^ T cells. The resulting expanded, effector-differentiated CD8^+^ T cells migrate to the site of infection to control and eliminate the pathogen. A fraction of activated antigen-specific CD8^+^ T cells give rise to central (Tcm), effector (Tem), and tissue-resident (Trm) memory T cells. Each of these memory CD8^+^ T cell subset possesses distinct migratory, phenotypic, and functional properties^17–21^ (see also ref.^16^).

A delay to generate effector CD8^+^ T cells or to recall circulating Tem to the site of infection allows the pathogen to replicate and cause tissue damage^22^. This damage can be minimized by vaccination that favours the establishment of local adaptive immunity for immediate recall to challenge^22^. Accordingly, studies of skin, intestine, female reproductive tract, and respiratory tract infections support the general concept that non-recirculating CD8^+^ Trm are optimally positioned at the barriers to confer protective immunity^1–12, 15, 22–24^ (see also ref.^16^). Notwithstanding, the need for an intranasal vaccine against respiratory infections remains unmet. This unmet need was particularly telling in a recent mechanistic study which showed that breakthrough infections occurred in individuals that did not install Trm cells even after one or two booster vaccination/s against SARS CoV2 via the intramuscular route^22, 25^.

Hence, insights into how Trm cells are generated, targeted to non-lymphoid tissues, and recalled to act as needed, will facilitate the development of novel vaccines/therapies against infectious diseases^17, 20^. To meet this goal, we recently reported the characterization of numerous HLA class I (HLA-I)-restricted, vaccinia virus (VAVC)-derived CD8^+^ T cell epitopes by using HLA-A*02:01 and B*07:02 (B0702^tg^) transgenic mice as models, which allow translational studies^26–29^. Several of these CD8^+^ T cell epitopes were also recognised by human volunteers vaccinated with VACV against smallpox^29^. The common mouse and human CD8^+^ T cell epitopes conferred protection against lethal respiratory infection of mice with VACV when delivered intravenously either as peptides in a TriVax regimen or as a protein subunit containing the epitope^15, 29^. Protection in this model required the installation of lung interstitium-resident CD8^+^ Trm cells^15, 29^.

Aerosol spread of pathogens has been a major cause of major past and recent endemics and pandemics. Hence, the respiratory mucosa is a vulnerable site of pathogen entry requiring constant immune surveillance^30^. Severe morbidity and mortality in respiratory infectious diseases are associated with virus dissemination and inflammation, which can damage the lung parenchyma and alveoli^29, 31–38^. Within the complex architecture of the lung, immune cells distribute to distinct anatomical compartments^39–43^. To protect from disease, memory CD8^+^ T cells populate different anatomical compartments of the lung that depend on the route of infection or vaccination^8, 15, 24, 42, 44, 45^.

MHC diversity is a major barrier for T cell targeted vaccine design. As mouse and human T cell epitopes differ because of trans-species MHC diversity, several groups have developed HLA-I^tg^ mouse as a model for epitope discovery and protection studies^15, 26–29,46^. We focused on B0702^tg^ mice because it is a prototypical member of the HLA-B7 supertype, which consists of HLA-I alleles accounting for ~ 0.45 in allele frequency^15, 46–48,29^. The logic was that lessons learnt from such a model is translatable to human vaccine development as well as ex vivo T cell response tracking and characterisation in vaccinees. Thus, for translatability, we adopted the B0702^tg^ mouse model in the past epitope discovery and protection studies^15, 29, 46^. Using this model, we previously reported two distinct microbe-free, protein-based immunogens (subunit vaccines) that elicit a robust pathogen-specific CD8^+^ T cell response^15, 29, 46^. For those afore reasons and because the current studies are a natural extension of our past works, B0702^tg^ mouse remained the model of choice.

The two protein subunits contain VACV-derived epitopes, which are recognised by both VACV and ectromelia virus-specific CD8^+^ T cells. Both vaccines elicit CD8^+^ T cells that localize to the lung vasculature, interstitium, and the airways^15, 46^. The interstitial CD8^+^ Trm cells are highly protective as it keeps pathogen burden in the lungs low as early as day one after challenge by the intranasal route^15^. Whether the protective interstitial CD8^+^ Trm cells are like those elicited by microbes in a real infection or by live, attenuated pathogens in conventional vaccination regimens remains unexplored. We found, first, the molecular signature of the subunit vaccine-elicited lung interstitial CD8^+^ Trm cells was distinct from subunit vaccine-elicited CD8^+^ Tem cells and splenic memory T (Tm) cells. Second, the transcriptome signature of subunit vaccine elicited CD8^+^ Trm cells, for most part, resembled those elicited by virus infection or vaccination. Lastly, the transcripts that code for chemokines and chemokine receptors, purinergic receptors, and adhesins lent clues to the basis of tissue residence and the quick action of vaccine-specific CD8^+^ Trm cells installed in the lung interstitium. These findings lend support for the development of subunit vaccines that can be safely delivered by the intranasal route.

## Results and discussion

### Intranasal instillation of a subunit vaccine installs CD8^+^ Trm in the lungs

**Figure 1:**
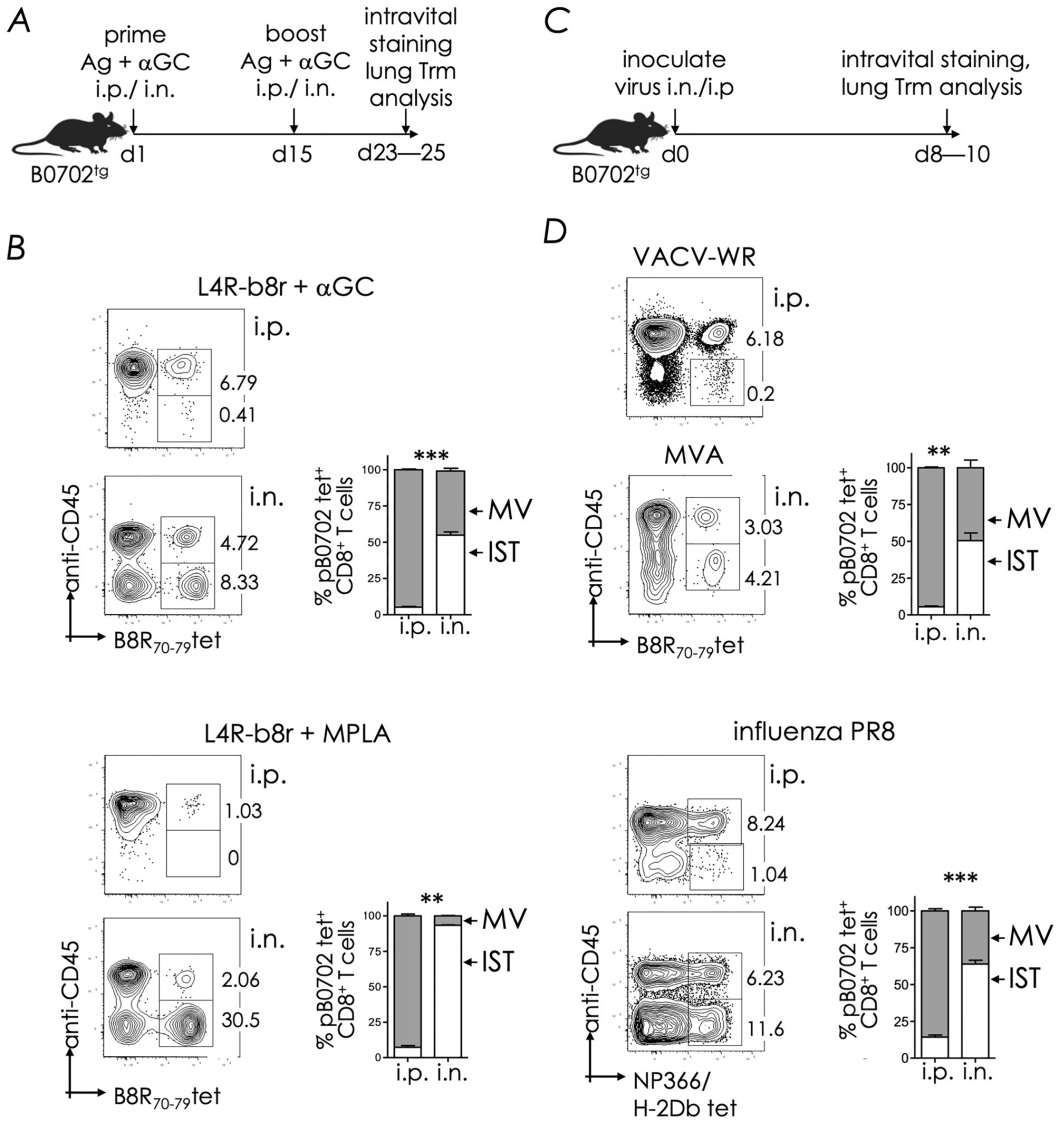
Distribution of immune CD8^+^ T cells in distinct lung compartments. (**A**) Experimental design: Mice were prime boost immunized i.p. or i.n. with L4R-b8r and αGC. On the day of harvest, mice were injected i.v. with anti-CD45.2-APC and euthanized after 5 min to allow intravital staining of circulating leukocytes. (**B**) Lungs were harvested and relative abundance of CD8^+^ Tem (marginated vasculature, MV) and Trm (interstitial, IST) cells in lungs based on the frequency of staining with anti-CD45.2-APC and B8R_70—78_/B0702 tetramer-PE. Plots are gated on viable CD8^+^ T cells. Bar graphs show relative ratio of Tem and Trm. (**C**) Experimental design: Mice were inoculated i.p. or i.n. route with the virus indicated in panel (**D**). After 8—10d post-inoculation (p.i.), mice were injected i.v. with anti-CD45.2-APC, and euthanized after 5 min. **D.** Lungs were harvested and analysed as in panel (**B**). Cumulative data from 2—3 independent experiments (*n* = 3–12 mice/group), mean ± SEM.

Prime boost vaccination of B0702^tg^ mice (which do not express the mouse H-2K^b^ & H-2D^b^) with protein antigen L4R-b8r mixed with the NKT cell agonist α-galactosylceramide (αGC) by the intranasal (i.n.) but not intraperitoneal (i.p.) route installs Trm cells in the interstitium (IST) and airways of the lungs^15^. L4R-b8r is a recombinant protein in which the non-protective, HLA-B*07:02 (B0702)-restricted L4R_37—45_ epitope was replaced with the protective, B0702-restricted B8R_70—78_ epitope^15^. We compared the magnitude of the response to αGC-adjuvanted L4R-b8r, first, to the response to the same antigen L4R-b8r but mixed with the toll-like receptor 4 ligand, monophosphorylated lipid A (MPLA) as the adjuvant in a prime boost regimen by both i.n. and i.p. routes (Fig. 1A). The magnitude of the CD8^+^ T cell response in the lungs to B8R_70—78_ epitope within the MPLA-adjuvanted L4R-b8r antigen was much higher than the response to the same epitope elicited by the αGC-adjuvanted antigen (Fig. 1B; compare top left & right panels). Both adjuvants elicited CD8^+^ IST Trm cells only when vaccinated by the i.n. route but very poorly when vaccinated by the i.p. route (Fig. 1B). Further the magnitude of the IST Trm response elicited by the MPLA-adjuvanted antigen was strikingly higher than those elicited by the αGC-adjuvanted antigen (Fig. 1B; compare left & right top panels). We conclude that the B8R_70—78_ is processed and presented by B0702 when immunised by both i.n. and i.p. routes irrespective of the adjuvant used.

We next compared the CD8^+^ T cell responses elicited by subunit vaccines to those elicited against viruses. Whilst the subunit vaccine was delivered to B0702^tg^ mice in a prime boost regimen, vaccina virus Western Reserve strain (VACV WR), Modified Vaccina Ankara (MVA), and influenza A virus (IAV) Puerto Rico (PR) strain were delivered to mice in a prime only regimen by i.n. (MVA, IAV) and/or i.p. (VACV, IAV) inoculation (Fig. 1C). These viruses were tested because they exemplify highly virulent (VACV WR), moderately virulent (mouse-adapted IAV PR), and avirulent, poorly-replicative (MVA) viruses. Results showed that i.n. MVA inoculation installed IST CD8^+^ Trm at a frequency comparable to that elicited by i.n. prime boost vaccination with αGC-adjuvanted L4R-b8r (Fig. 1B and D; compare the left top panel with the right bottom panels). The response to i.n. MVA inoculation was weaker than the response to i.n. IAV inoculation, and the same was with i.p. inoculation (Fig. 1D; compare top & bottom panels). Likewise, the response to i.n. prime boost vaccination with αGC-adjuvanted L4R-b8r was weaker than the response to i.n. IAV inoculation (Fig. 1B; compare left top & right bottom panels). Vaccination with adjuvanted antigens and inoculation with VACV and IAV via the i.p. route resulted a very low frequency of IST CD8^+^ T cells (Fig. 1B and D).

**Figure 2:**
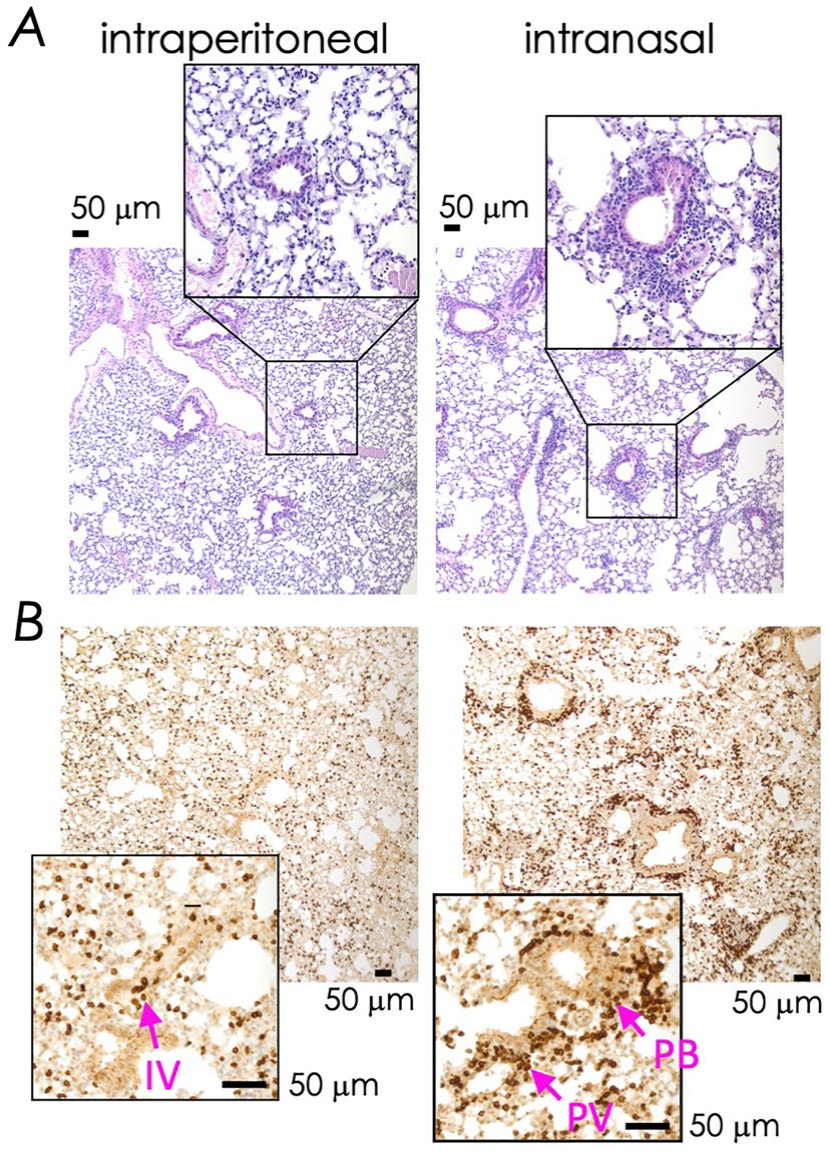
Comparative histologic and immunohistologic localisation of leukocytes responding to subunit vaccination and virus infection. (**A**) Mice were prime boost immunized i.p. or i.n. with L4R-b8r and αGC and lungs were harvested on d14 post boost. H&E staining of lungs from i.n. immunized mice showed prominent peri-vascular and peri-bronchiolar inflammatory infiltrate (lymphocytes and macrophages) compared to lungs of IP immunized mice. Data represent one of 3 lung sections per mouse from 3 mice per group. (**B**) Anti-CD3 staining of lung sections performed on d10—14 after booster immunization by i.p. or i.n. route as in Fig. 1. Arrows designate peri-vascular (PV), peri-bronchiolar (PB) and intravascular (IV) T cells. Scale bars, 50 μm. Data are representative of four sections per lung of two mice from two independent experiments per condition.

To localise CD8^+^ Trm cells so elicited, we performed histological and immunohistochemical analyses of the lungs after the same prime boost vaccination with αGC-adjuvanted L4R-b8r antigen as described above (Fig. 1A). Two weeks after i.p. vaccination, we found intravascular (IV) infiltration of mononuclear cells, some of which were CD3 positive (Figs. 2A and B, left panels). By contrast, i.n. vaccination resulted in perivascular (PV) and peribronchiolar (PB) localisation of mononuclear cells, some of which were CD3 positive (Fig. 2A and B, right panels). Taken together we conclude that microbe-free subunit vaccines elicit CD8^+^ T cell responses similar to virus infections, and that efficient Trm cell installation depends on the route of antigen exposure—i.n. route being much more efficient than the i.p. route, as reported previously^15^.

### Prime boost vaccination with rOVA-3 vaccine elicits lung CD8^+^ Trm response

**Figure 3:**
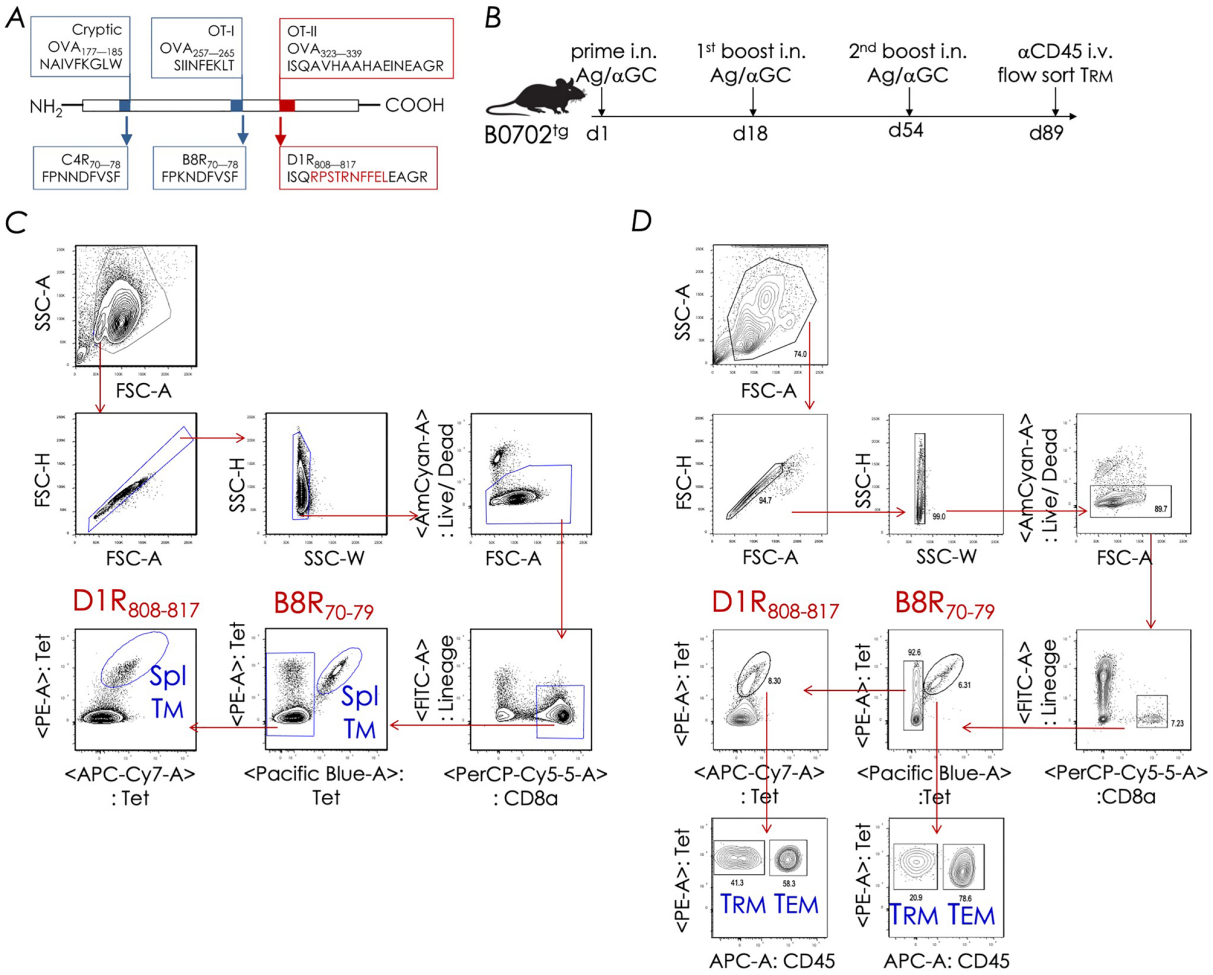
Prime boost vaccination with rOVA-3 elicits B8R_70–78_ and D1R_808–817_-reactive CD8^+^ T cells. (**A**) Diagram showing OVA (rOVA-3) construct in which the original cryptic, OT-I and OT-II epitopes were replaced with C4R_70–78_, B8R_70–78_ and D1R_808—817_ epitopes, respectively. A six-histidine tag at the C-terminus of rOVA-3 facilitated purification after expression in *E. coli*. (**B**) Mice were primed with rOVA-3 + αGC, boosted twice with the same vaccine, and CD8^+^ T cells isolated and purified on days shown. (**C&D**) Intravital staining was performed as in Fig. 1, and B8R_70–78_/B0702 and D1R_808—817_/B0702 tetramer-reactive cells were purified by FACS using the gating strategy shown for splenic (**C**) and pulmonary (**D**) CD8^+^ T cells. Purified pB0702 tetramer-reactive cells were used for downstream transcriptomic studies (*n* = 12 mice; each replicate consisted of cells pooled from 3 mice).

Natural pulmonary infections and intranasal vaccinations with attenuated pathogens elicit highly protective CD8^+^ Trm responses (reviewed in ref.^16^). Such protective CD8^+^ Trm cells in mice and humans are characterised by a lineage-specific gene-regulatory network^3, 49–51^ (see also ref.^16^). As a means to purify large numbers of protective CD8^+^ Trm cells, we recently reported the construction and use of a recombinant ovalbumin (rOVA) in which all three T cell epitopes in OVA were replaced with D1R_808—817_ [in place of H-2K^b^-restricted OT-I (Ova_257—264_)], B8R_70—78_ [replacing nine amino acids of H-2A^b^-restricted OT-II (Ova_323—339_)], and C4R_70—78_ [substituting the cryptic H-2D^b^-restricted epitope (Ova_177—185_)] resulting in recombinant rOVA-3 (Fig. 3A).

Intranasal prime boost vaccination with rOVA-3 mixed with αGC^15, 29, 52, 53^, as shown in Figure 3B, resulted in a B8R_70—78_ and D1R_808—817_ reactive CD8^+^ T cells both in the lungs and the spleen (Figs. 3C and D). Because OVA-3 lacks the natural CD4^+^ T cell epitope—OT-II, and as MPLA adjuvancy requires a functional CD4^+^ T cell response^54^, the studies described below were performed using αGC as the adjuvant. Moreover, B8R_70—78_ and D1R_808—817_ reactive CD8^+^ T cells in the lungs contained both anti-CD45^neg^ IST Trm cells as well as anti-CD45^pos^ MV CD8^+^ T cells (Fig. 3D). We previously reported that these B8R_70—78_ and D1R_808—817_ reactive CD8^+^ T cells had features of IST Trm and MV Tem cells as well as splenic Tm cells^46^. Hence, we have retained this memory nomenclature and use them as operational terms in the rest of this report. The three memory CD8^+^ T cell subsets were flow sorted after enrichment for studies described below.

### Lung IST CD8^+^ T cells express a transcriptomic signature characteristic of Trm cells

Next, we defined the molecular signature of lung-resident IST CD8^+^ Trm cells and compared it to that of lung MV Tem and splenic Tm cells that were induced by subunit vaccination. Hence, the transcriptome of both B8R_70—78_ and D1R_808—817_ reactive IST and MV CD8^+^ T cells as well as splenic CD8^+^ Tm cells were characterised by population level RNA sequence (RNAseq) determination in four replicates (see Table S1) by using next generation technologies and platforms. Our previous studies showed that the expression of CXCR3, CD69, and CD103 by subunit vaccination-induced lung IST Trm cells was either low (CXCR3), shared by a fraction of Tem cells (CD69), or not on all cells (CD103)^15, 46^. Further, a recent study showed the CD69 expression was dispensable for lung tissue residence^55^. Hence, unalike other reports, these markers were not used to purify the different memory CD8^+^ T cells subsets for this study. But instead, circulating leukocytes in rOVA-3 prime boost vaccinated mice (see Fig. 3B) were intravitally stained with anti-CD45-APC for 3–5 min^15, 46^. Lung B8R_70—78_ and D1R_808—817_ pB0702 tetramer reactive CD8^+^ T cells were flow sorted into anti-CD45-APC^neg^ IST Trm and anti-CD45-APC^pos^ MV Tem cells. From the same mouse group, splenic pB0702 tetramer reactive Tm cells were also flow sorted as above except that these cells were not separated into anti-CD45-APC^pos^ and anti-CD45-APC^neg^ fractions. The purity of sorted lung IST Trm and MV Tem, and splenic Tm cells was > 95% (data not shown). B8R_70–78_ and D1R_808–817_ reactive CD8^+^ T cells from each compartment and from the same replicate were pooled so that each of the IST Trm, MV Tem, and splenic Tm subsets yielded ~ 4–5 × 10^5^ cells (Table S1), from which RNA was isolated and processed for next generation RNAseq experiment.

We experimentally validated the key features of each of the purified CD8^+^ T cell memory subset by flow cytometry. Lung IST Trm cells distinguished themselves from the other two memory subsets as they showed a CD103^HI^ & CD103^LO^, CD69^+^, CXCR6^+^, CXCR3^LO/NEG^, CD62L^LO^, KLRG1^LO^ phenotype. By contrast, MV Tem cells were CD103^NEG^, CD69^NEG^, CXCR6^INT^, CXCR3^LO/NEG^, CD62L^LO^, KLRG1^HI^ and splenic Tm cells were CD103^NEG^, CD69^NEG^, CXCR6^INT^, CXCR3^INT^, CD62L^HI^, KLRG1^LO/NEG^. These data, which emerged as a part of this study, were reported previously by us^46^ and not reported here. Hence, the differences described herein are at the mRNA level.

**Figure 4:**
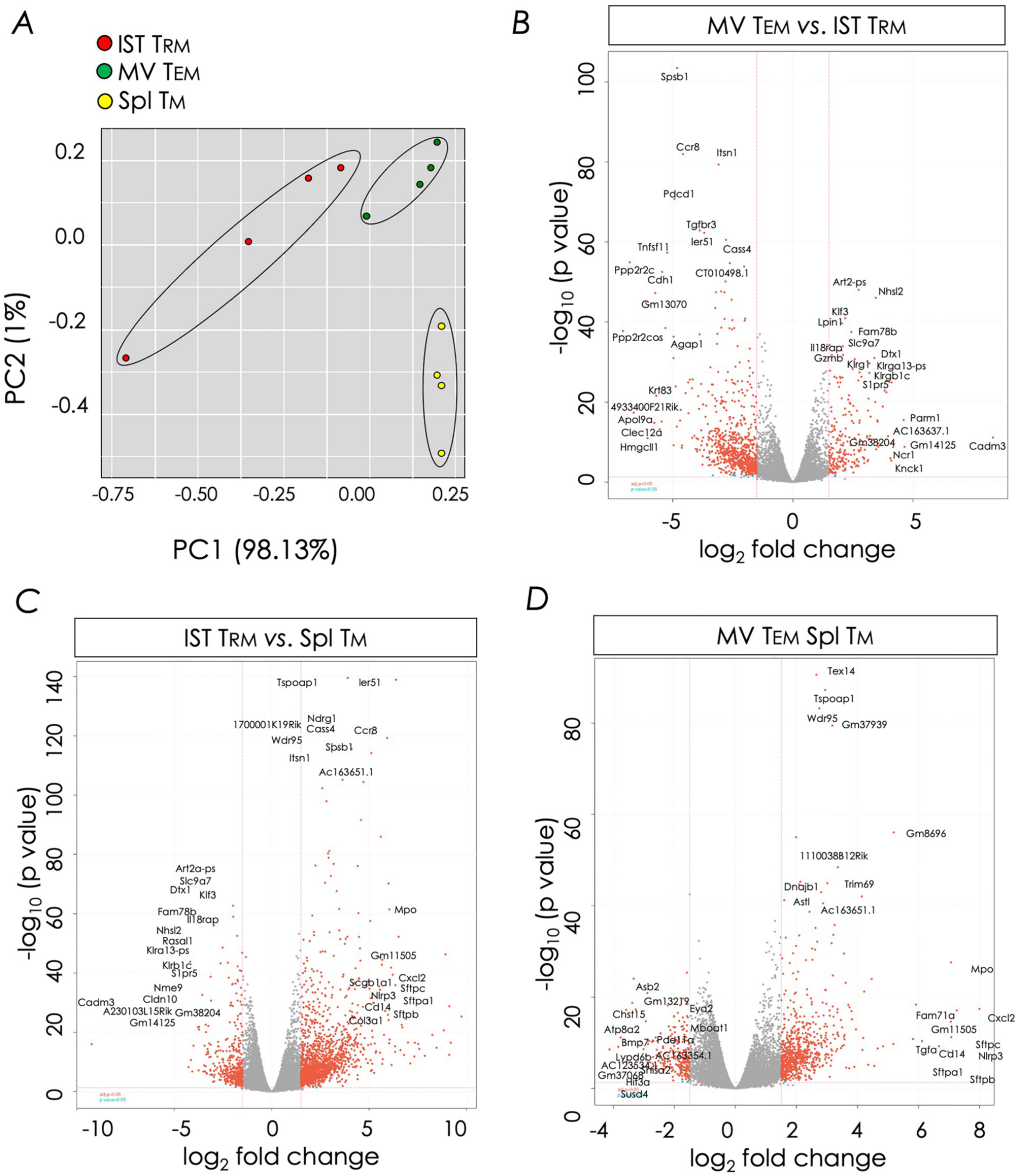
Distinct transcriptome signatures define CD8^+^ lung IST Trm, MV Tem, and splenic Tm cells. (**A**) Principal component analysis of CD8^+^ IST Trm, MV Tem, and splenic Tm transcriptomes. (**B–D**) Volcano plots showing log_2_ fold change against -log_10_ adjusted *p* value of approximately 17,000 transcripts. Top 20 up and down regulated genes are shown in the volcano plots.

Principal component analysis and hierarchical clustering of the transcriptome data showed that the three CD8^+^ T cells—IST Trm, MV Tem, and splenic Tm, formed distinct subsets. Further, as expected, the lung IST Trm and MV Tem were closely related to each other but only distantly related to the splenic Tm subset (Fig. 4A). Supporting the principal component analysis—pairwise hierarchical clustering of the RNAseq-derived transcriptome data, using a threshold of log_2_ fold change of 2 and adjusted *p* ≤ 0.05, also showed that CD8^+^ IST Trm, MV Tem, and splenic Tm cells segregated into distinct subsets (Figs. 4B–D and S1).

To identify pathways that govern IST Trm identity and function, gene ontology-based gene set enrichment analysis (GSEA) was performed by pairwise comparisons of gene expression between IST Trm *versus* MV Tem, IST Trm *versus* splenic Tm, and MV Tem *versus* splenic Tm. This comparative gene expression analyses showed significant differences between the three CD8^+^ T cell memory subsets. Major differences between the three CD8^+^ T cell memory subsets were in genes that control G-protein coupled receptor activity, signalling and chemotaxis, cell adhesion and locomotion, transmembrane receptor activity and solute transport, T cell co-stimulation and signalling, cytokine responses and TNF pathway signalling, and inflammation (Tables S2–S4). These altered pathways are similar, albeit not identical to those reported previously for CD103^+^ Trm cells when compared to CD103-negative Tem cells in human lungs^49, 56^. It should be noted that the lung IST Trm used in this study contained a mixture of CD103^+^ and CD103^LO/NEG^ cells^46^, which may in part explain some of the noted differences. Additionally, some of the differences perhaps relate to history of past and recent exposures to pathogens and to the types of microbes of human lungs when compared to the relative low to no exposure of the laboratory mouse lungs to microbes.

**Figure 5:**
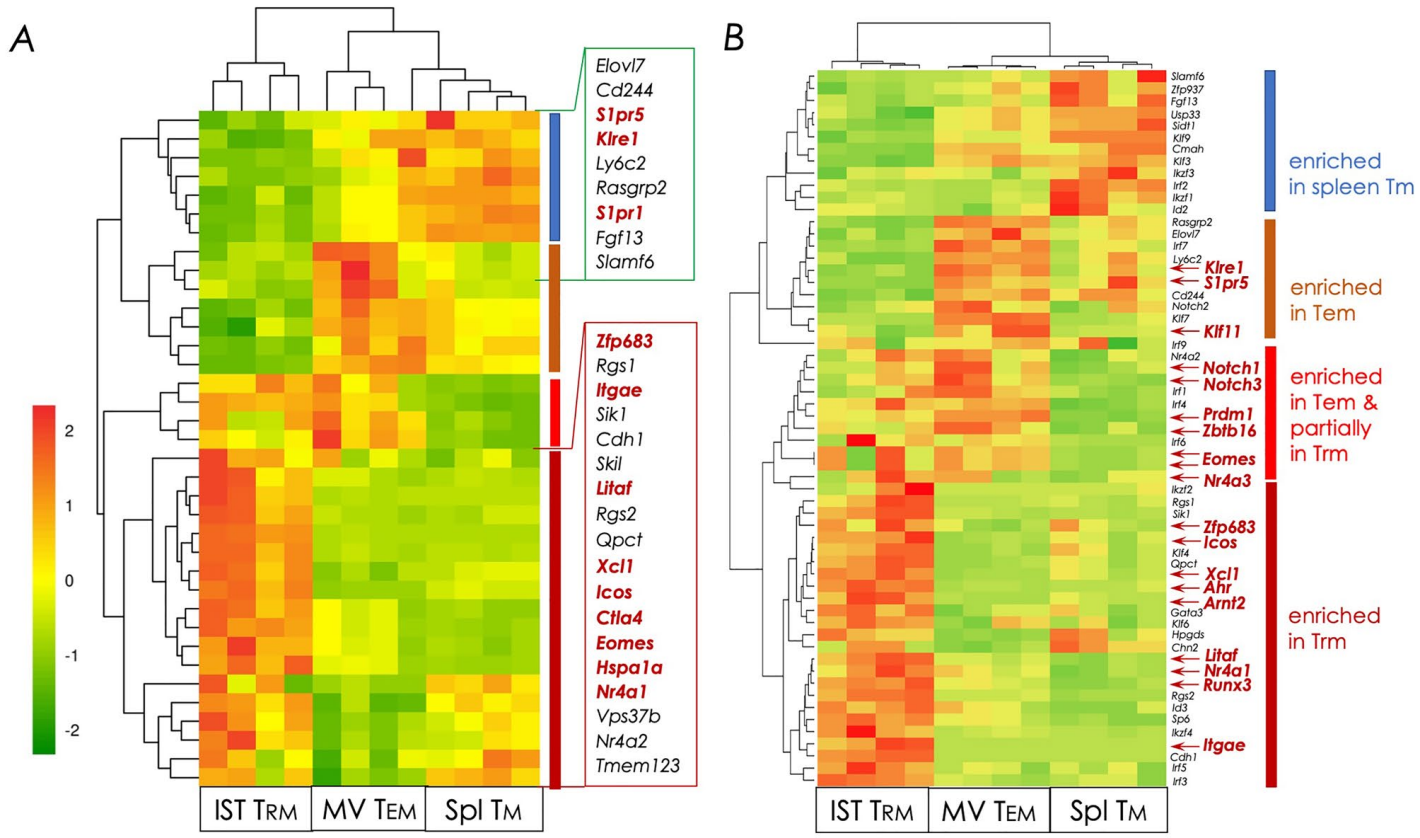
Heat map representation of Trm signature genes. Clusters of differentially expressed transcripts within CD8^+^ IST Trm, MV Tem, and splenic Tm cell subset were ordered by K-means clustering analysis as described in *Materials and Methods*. Bulk RNAseq data derived from the three CD8^+^ memory T cell subsets were used for this analysis. (**A**) Differential expression of thirty-six previously reported Trm cell signature genes^3^ wherein log_2_ fold change ≥1.5 with adjusted* p* ≤ 0.05 are shown. Blue side bar, splenic Tm cell-; brown side bar, lung MV Tem cell-; red sidebar, common MV Tem & lung IST Trm-; and maroon sidebar, IST Trm-specific gene expression pattern. (**B**) Differential expression analysis of transcriptional factors along with Trm signature genes^3, 49^ were performed as in (**A**) using bulk RNAseq data derived from the three memory T cell subsets. Highlighted are differentially expressed transcripts with log_2_ fold change ≥1.2 with adjusted *p* ≤ 0.05 are shown. Sidebars are the same as in (**A**).

**Figure 6:**
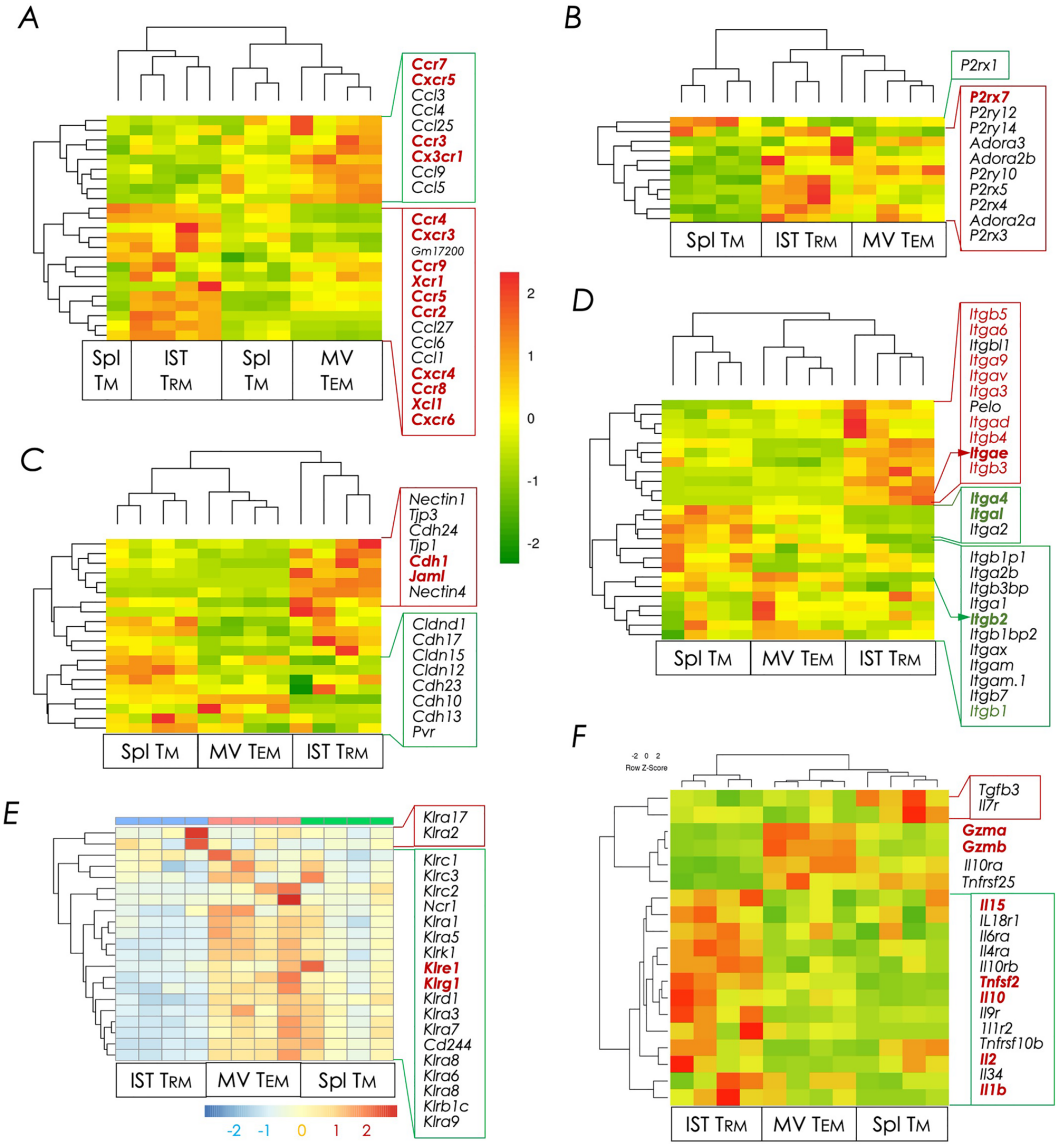
Heat map representation of pathway-specific gene expression changes. Clusters of differentially expressed transcripts specific within the indicated pathways (**A–F**) were ordered by K-means clustering analysis as described in *Materials and Methods*. Bulk RNAseq data derived from CD8^+^ IST Trm, MV Tem, and splenic Tm cell subsets were used for this analysis. (**A**) Chemokines and chemokine receptors. (**B**) Integrins. (**C**) Cell adhesion molecules. (**D**) Purinergic receptors. (**E**) inhibitory & activating killer cell lectin-like receptors. (**F**) T cell effector molecules.

We identified 1985 unique genes that were differently expressed: 1,566 genes were up-regulated and 419 down-regulated when CD8^+^ IST Trm cells were compared to splenic Tm cells. Similarly, MV Tem cells differently expressed 981 unique genes, of which 739 were up-regulated and 242 down-regulated when compared to splenic Tm cells. So also, Tem cells differently expressed 1019 unique genes, with 233 genes up-regulated and 786 down-regulated, when compared to Trm cells (Fig. 4B–D and S1**)**. Among the differentially expressed genes were Trm-lineage specific master regulators and their target genes. The latter included genes that are known adhesins and effector molecules such as cytokines and chemokines, and their receptors, purinergic receptors, and killer-like lectin receptors (Figs. 5 and 6). These differentially expressed genes and their known mechanism/s in T cells are discussed below.

### Subunit vaccination-induced CD8^+^ Trm cells express lineage-specific transcription factors

Comparison of CD8^+^ IST Trm, MV Tem, and splenic Tm transcriptomes activated by subunit vaccination revealed several previously reported genes expressed (adjusted *p* ≤ 0.05) only within IST Trm but not in MV Tem and splenic Tm cells. Whilst several MV Tem- and splenic Tm-specific genes were upregulated, a subset of genes were expressed commonly within MV Tem cells and in IST Trm cells as well, albeit at a low level (Fig. 5). The commonly expressed genes may suggest the relatedness of IST Trm and MV Tem cells observed upon principal component analysis of the three transcriptomes (Fig. 4A).

Among genes differentially expressed by log_2_ 1.5 fold change (adjusted *p* ≤ 0.05) in expression were IST Trm-specific genes which included the master transcription factor *Zfp683* encoding Hobit, the prototypic marker for tissue-resident leukocytes *Itgae* coding for CD103, as well as cytokine *Litaf*—which encodes lipopolysaccharide-induced tumor necrosis factor, and chemokine *Xcl1*, and CD8^+^ T cell activation induced *Icos, Ctla4*, *Eomes*, *Hspa1a*, and *Nr4a1* genes (Fig. 5A). Expression of *Icos*, which encodes inducible T cell costimulator (ICOS) in IST Trm cells is consistent with the recent report that signalling via ICOS generates Trm cells^57^.

TGFβ signalling is essential for CD103^+^ CD8^+^ Trm cell differentiation and the induction of the tissue residence program. In response to TGFβ signalling, CD103^+^ CD8^+^ Trm cells in various non-lymphoid tissues, including the lungs, shut-off *Eomes* expression, which codes for the T-box transcription factor eomesodermin^58^. We previously reported that lung IST Trm cells elicited by subunit vaccination were composed of both CD103^+^ and CD103^LO/NEG^ cells^15, 46^ as has been noted by others in response to virus infection as well^13, 59, 60^. CD103^LO/NEG^ Trm cells poorly express TGFβ receptor and, hence, are unresponsive to TGFβ^60^. The presence of CD103^LO^ cells in the lung IST Trm subset in this study may in part explain *Eomes* expression by lung IST Trm cells reported herein.

Further inspection of the RNAseq data revealed that the *S1pr1* and *S1pr5* genes coding for sphingosine-1-phosphate receptor (S1PR) 1 and 5 were down regulated in IST Trm and MV Tem cells but were highly expressed in splenic Tm cells (Fig. 5A). *S1pr1* and *S1pr5* downregulation is essential for T cell egress from secondary lymphoid organs, especially the lymph nodes—the site of primary T cell priming, and traffic to take residence in non-lymphoid tissues such as the lung interstitium^61, 62^. Overall, the observed Trm-specific gene expression identified several key transcripts but not all of them that typify tissue residence of memory CD8^+^ T cells.

Therefore, transcripts from the top 60 genes with log_2_ 1.2-fold change (adjusted *p* ≤ 0.05) in expression between IST Trm, MV Tem, and splenic Tm were identified. This resulted in several other differentially expressed genes, most notably the master transcriptional regulator *Runx3* (Fig. 5B). Runx3 controls CD8^+^ T cell thymocyte development and cytotoxic activity of mature CD8^+^ T cells^63–66^. Accordingly, it is expressed by Trm cells and is required for Trm development and function^66, 67^. Whilst *Runx3* expression in circulating memory CD8^+^ T cells was previously reported^66^, it is not expressed in lung MV Tem cells (Fig. 5B). The reason for this difference in *Runx3* expression in the two studies may have resulted from the use of monoclonal^66^ *versus* polyclonal (this study) CD8^+^ T cells and/or virus^66^ *versus* subunit vaccination (this study). Understanding the cause will require further investigation, however.

*Pdrm1*, which codes for Blimp-1—a transcription factor expressed by Trm cells homing to select non-lymphoid tissues^68^, was expressed by IST Trm and MV Tem cells of the lungs (Fig. 5B). Blimp-1 by cooperating with Hobit, which is not expressed by MV Tem cells, represses *Klf2* (not observed in our dataset), *S1pr1* (Fig. 5), and *Ccr7* (see Fig. 6C) to promote CD8^+^ T cell residence in the lungs^3, 50, 51, 56, 66, 69, 70^.

Additional genes that control tissue residence and maintenance of CD8^+^ cells^50, 56, 69^ were also enriched within subunit vaccination-induced IST Trm cells. These included *Ahr*, which codes for arylhydrocarbon receptor (AHR), and AHR signalling pathway gene *Arnt2*, as well as *Nr4a1*, which codes for Nur77—a pleiotropic transcription factor induced downstream of T cell receptor activation. *Notch* genes were expressed in human lung CD103^+^ Trm cells^49^ and implicated in maintenance and metabolism of these cells. *Notch* genes were not specifically expressed in subunit vaccination-induced IST Trm cells but were upregulated in MV Tem cells at a level higher than by Trm cells and were absent in splenic Tm cells (Fig. 5B). Furthermore, a comparison of gene expression between CD8^+^ T cell memory subsets resulting from intranasal subunit prime-boost vaccination and virus infection of mice^56^ showed significant similarities (Fig. S2). Collectively therefore, the data suggest protein subunit vaccination induces key transcription factors and target genes essential for the generation, installation, and maintenance of CD8^+^ Trm cells in the lungs.

### Lung IST Trm cells express a distinct chemokine receptor repertoire

Specific chemokines and their receptors play critical roles in the egress of activated T cells from the site of priming and migration to the site of inflammatory insult. Hence, we next determined subunit vaccination-induced chemokine and chemokine receptors in lung IST Trm and MV Tem cells as well as splenic Tm cells. A distinct IST Trm and MV Tem chemokine receptor signature was evident, but no splenic Tm specific signature was readily seen. Thus, IST Trm but not MV Tem cells expressed the transcript for CXCR3 (Fig. 6A) even though the former cells do not express the protein on the cell surface^15, 46^. IST Trm cell preparation may have contained CXCR3^HI^ airway Trm cells^15, 46^, which were not depleted, which may explain *Cxcr3* expression (Fig. 6A).

*Cxcr6*, *Xcr1*, and *Ccr5* transcripts distinguished IST Trm cells from MV Tem cells. We previously reported the expression of CXCR6 in subunit vaccination-induced IST Trm cells^46^. Others have reported the expression of CXCR6 and CCR5 by mouse Trm cells as well as human CD103^+^ lung Trm cells, and elucidated the importance of CXCR6 in lung homing^49, 71^. On the other hand, MV Tem cells distinguished themselves by higher expression of *Ccr7*, *Ccr3*, *Cxcr5*, and *Cx3cr1* transcripts over IST Trm cells (Fig. 6A). Other studies have shown the expression of the fractalkine (CX3CL1) receptor CX3CR1 on the surface of human peripheral blood Tem cells. As CX3CR1 promoted trans-endothelial migration of leukocytes^72^, its expression on MV Tem cells suggested these cells were poised for migration across the capillary endothelium into the lung interstitium. Overall, our data suggest a lineage specific chemokine receptor expression pattern that may have driven subunit vaccine-activated Trm cells to the lung interstitium.

Recent evidence suggests that extracellular ATP (adenosine triphosphate)- and NAD^+^ (nicotinamide adenine dinucleotide)-sensing purinergic receptor P2XR7 controls metabolic fitness of T cell to maintain long-term immunologic memory and plays an essential role in CD8^+^ Trm generation in mouse small intestine and lungs^73–76^. Hence, we determined the expression pattern of purinergic receptors by CD8^+^ IST Trm, MV Tem cells and splenic Tm cells generated by subunit vaccination. We found significant expression of *P2xr7* by IST Trm cells and modest expression by splenic Tm cells. MV Tem cells expressed little if any *P2xr7* (Fig. 6B). The expression of P2XR7 by lung-resident Trm cells may partly explain why they quickly wane from the lungs^77^, wherein ATP concentration may be high due to environmental and hyperoxygenic stresses.

### Effector molecules of tissue residence

The interplay of integrins and cadherins facilitate migration of lymphocytes to the site of inflammation and tissue residence. Hence, the expression of genes that code for such molecules were ascertained. We found higher *Itgae* expression in IST Trm cells when compared to MV Tem and splenic Tm cells (Figs. 5 and 6D). *Itgae*—a gene controlled by Runx3, and Notch1 and Notch2, codes for integrin αE/CD103—the signature tissue-residence marker for many leukocytes including CD8^+^ Trm cells^49, 50, 78, 79^. Even though we found Notch 1 and Notch3 expression in both IST Trm and MV Tem cells, the latter do not express Runx3 (Fig. 5B), which may explain the lack of *Igtae* expression in MV Tem cells (Figs. 5 and 6D). Functionally, αE pairs with the integrin β7 to form the receptor for E-cadherin (encoded by *Cdh1*). αEβ7 mediates adhesion to epithelial cells and, hence, helps to retain them within tissues^79, 80^.

All T cells express LFA (lymphocyte function-associated antigen)-1—a heterodimer of integrins αL and β2—encoded respectively by *Itgal* and *Itgb2*. αLβ2 interacts with endothelial ICAM (intercellular adhesion molecules)-1 to enact immune surveillance of the vasculature^81^. Lung IST Trm cells express a very low level of other integrin genes such as *Itgb2, Itgal*, *Itga2*, and *Itga4* (Fig. 6D) and hence, do not express the αLβ2 integrin, consistent with its role in immune surveilling the vasculature. Surprisingly, however, lung MV Tem cells also lacked the expression of *Itgb2*, *Itgal*, *Itga2*, and *Itga4* genes (Fig. 6D). *Itgal*, *Itga4*, and *Itgb2* are Notch1- and Notch2-regulated genes^49^. Moreover, IST Trm and MV Tem cells, but not splenic Tm, express Notch 1 and Notch 3 (Fig. 5B). Hence, our results are inconsistent with the recent report which showed down regulated *Itgal*, *Itga4*, and *Itgb2* expression consequent to the inhibition of Notch signalling in established mouse lung CD69^+^ CD8^+^ Trm cells^49^. The reason for the contradictory data is unknown. A potential difference between the two studies is antigen persistence. IAV-derived NP antigen, the model used in the published study^49^, may persist longer than the subunit vaccine, which was perhaps quickly cleared from the lungs in our study.

Lung IST Trm cells selectively expressed *Itgav*, *Itgb3*, *Itga3*, *Itgb5*, *Itga6*, *Itgb4*, *Itga9*, and *Itgad*, but not *Itgb1 or Itgb2* (Fig. 6D). Integrin αVβ3 binds to fractalkine/CX3CL1 and may act as a coreceptor in CX3CR1-dependent fractalkine signalling^82^. As *Cx3cr1* is expressed by MV Tem cells, what role αVβ3-fractalkine interaction plays in lung residence is unknown. Moreover, *Itga3* and *Itgb5* encode integrins that form the heterodimeric VLA-3, the receptor for vitronectin expressed by tissues. α6β4 integrin is a receptor for laminin expressed on the surfaces of epithelial cells. These known T cell integrins and cognate epithelial cell ligand interactions perhaps promote and reinforce lung interstitial residence.

Marginated vascular Tem cells modestly expressed *Itgal* and a significant level of *Itgb2* (Fig. 6D), which together code for the αLβ2 integrin LFA-1. LFA-1 was previously reported to be expressed Nonetheless, human CD8^+^ T cells were recently shown to form a synapse with endothelial cells to diapedese when signaled by CX3CL1 and ICAM-1 —the receptor for LFA-1^83^. Liver Trm cells, which surveil the sinusoids by crawling on sinusoidal endothelium, also express LFA-1^84^. Consistent with the reported interactions of T cells with endothelial cells^83–85^, one might predict then, LFA-1 together with CX3CR1 make MV Tem cells receptive to danger signals emanating from the vascular endothelial cells and the local tissue umwelt to prompt diapedesis/trans-endothelial migration into tissue parenchyma^83, 86^—i.e., as related to this study, into the lung interstitium. This feature of MV Tem cells may also play a role not only in quick response to infectious threats, but also in homeostatic replenishment of waning IST Trm cells in the lungs^77^.

Other adhesins involved in cell–cell interactions that tether leukocytes to tissues are calcium-dependent cadherin (CDH) and junction adhesion molecule (JAM) family of proteins. Lung IST Trm cells express *Cdh1* that codes for CDH1 and *Jaml* which encodes JAM like (JAM-L) molecule (Fig. 6C). CDH1 function in T cells is unknown. CDH1 engage in homotypic interactions, which suggests the engagement of IST Trm cell CDH1 with lung interstitial epithelial CDH1 (also known as E-cadherin) may retain Trm cells within the tissue. Moreover, whilst JAMs are endothelial and epithelial cell-restricted adhesins, JAM-L is expressed solely by leukocytes. Leukocyte JAM-L homodimer promotes adhesion to epithelial cells via heterotypic interactions with coxsackie and adenovirus receptor (CXADR). In T cells, JAM-L interacts in *cis* with very late antigen 4—an integrin made of α4β1 and prevents JAM-L dimerization^87^. As IST Trm cells do not express *Itga4* and *Itgb1* (Fig. 6D), JAM-L are in a homodimeric state which perhaps make them receptive to interactions with epithelial CXADR to enforce tissue retention.

### Effectors of CD8^+^ T cell function

Activated CD8^+^ T cells are known to express natural killer cell receptors that belong to the killer cell lectin-like receptor (KLR) family of proteins. KLR family consists of Ly49, NKG2, and other stimulatory or inhibitory molecules. CD8^+^ T cells that express KLRs can function as innate-like cells, responding rapidly to an infection, control the magnitude and duration of cytotoxic responses, or prevent apoptosis and exhaustion of responding immune cells^88–95^. As lung, skin and female reproductive tract Trm cells respond quickly^15, 96–98^, akin to innate immune cells, the expression of *Klr* family of genes was interrogated. We found little evidence for the expression of *Klr* family of genes by lung CD8^+^ Trm cells elicited by subunit vaccination (Fig. 6E). Nonetheless, multiple different *Klr* genes were expressed by MV Tem cells, some of which were also expressed by splenic Tem cells. These included *Klrb1c* (NK1.1; generally stimulatory but inhibitory in some contexts), *Klrc1* (NKG2A; inhibitory), *Klrc2* (NKG2C, stimulatory), *Klrd1* (CD94; stimulatory or inhibitory), *Klre1* (NKG2I; stimulatory or inhibitory), *Klrg1* (KLRG1; inhibitory), and *Klrk1* (NKG2D; stimulatory) (Fig. 6E), which are known to control various functions of memory CD8^+^ T cells^88–95^.

To act quickly, MV Tem had upregulated *Gzma* and *Gzmb* genes, which code for the canonical cytotoxic molecules granzyme (Gzm) A and GzmB (Fig. 6F). By contrast, to maintain a controlled response, lung IST Trm expressed *Il10* and its receptor gene *Il10ra* (Fig. 6F). Taken together, the expression of both stimulatory and inhibitory KLR molecules by MV Tem cells may set the subtle balance between a quick immune response and the need to prevent immunopathology (disease tolerance;^99, 100^) to maintain homeostasis in the lungs.

### Prime boost vaccination with unadjuvanted rOVA-3 vaccine elicits a low magnitude CD8^+^ T cell response

Preparation of endotoxin-free OVA is a difficult task^101^. Furthermore, MPLA-adjuvanted L4R-b8r induces a strong CD8^+^ Trm response compared to the vaccine containing αGC as the adjuvant (Fig. 1B). Hence, the contribution of co-purifying adjuvants in rOVA-3 containing vaccine was ascertained.

**Figure 7:**
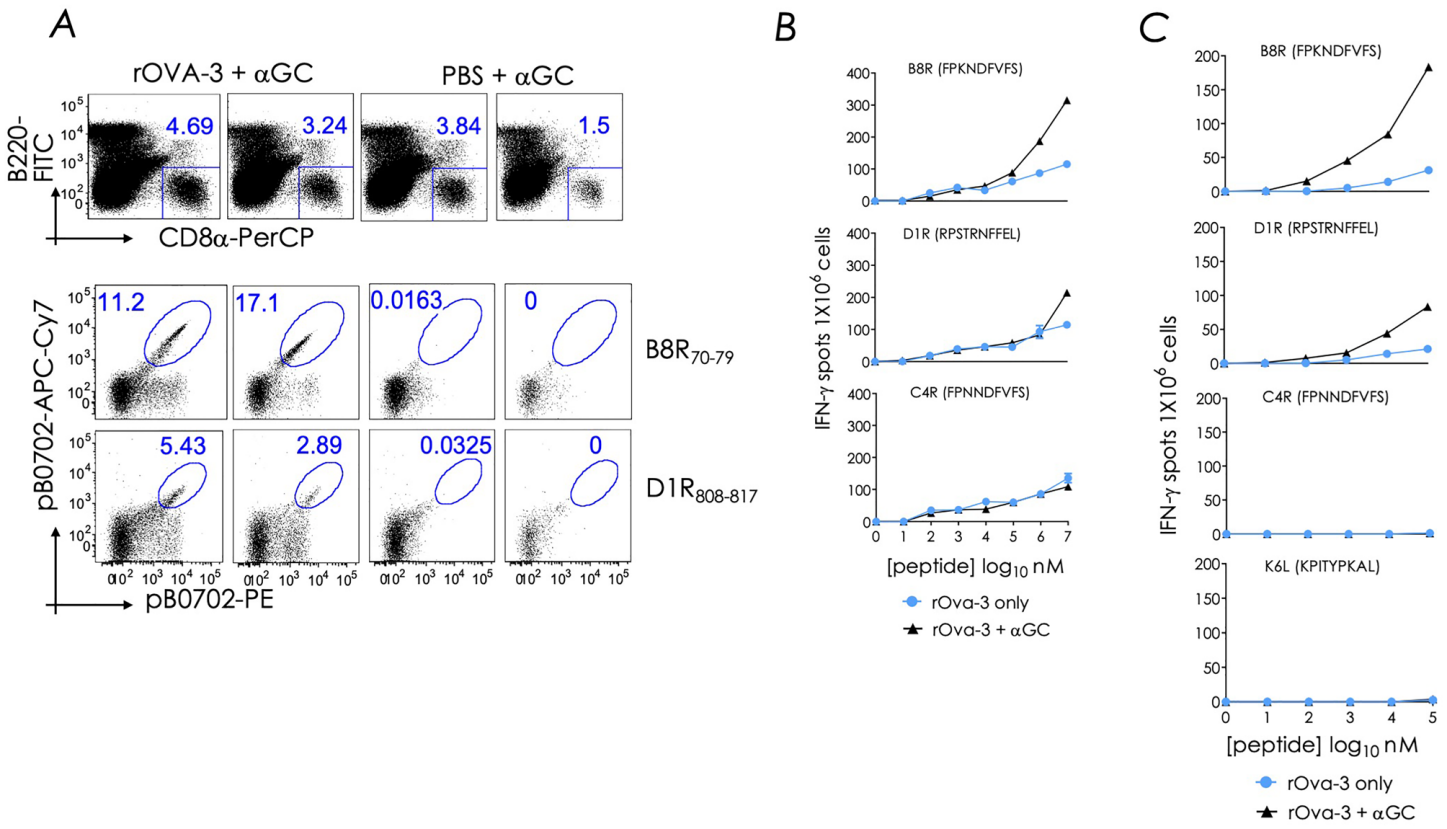
Prime boost vaccination with rOVA-3 without endotoxin depletion elicits a low but substantial level of B8R_70–78_ and D1R_808–817_-reactive CD8^+^ T cell response. (**A**) B0702^tg^ mice were primed and boosted by the i.n. route, two weeks apart with rOVA-3 mixed with αGC or with αGC alone. Lungs were collected d15 post boost. Cells were stained and gated on live CD8^+^ T cells and B8R_70–79_, D1R_808-817_ tetramer positive cells. Experiments were reproduced at least two times (*n* = 2 mice) and several times in our published report ^46^. (**B**) B0702^tg^ mice were primed and boosted as in (**A**). Endotoxins were not depleted from this rOVA-3 preparation used for vaccination. On d15, splenic T cell response to the indicated B0702-restricited epitopes were determined by measuring IFN-γ response in an ELISpot assay. Data represent the mean of triplicate wells; representative of one experiment; (*n* = 3 mice). (**C**) The experiment in (**B**) was repeated with endotoxin-depleted rOVA-3 and the response measured in an IFN-γ specific ELISpot assay using the indicated peptides. Data represent the mean of triplicate wells; representative of two independent experiments; *n* = 3 mice in each experiment.

Thus, B0702^tg^ mice were prime boost vaccinated with rOVA-3 mixed with αGC two weeks apart, and 18d later, B8R_70—78_/B0702 and D1R_808—817_/B0702 tetramer reactive CD8^+^ T cell responses in the lungs were monitored. Note that B0702^tg^ mice to not respond to C4R_70—78_^46^ and, hence, reactive CD8^+^ T cell response was not monitored. As expected^46^, we found that B0702^tg^ mice responded to B8R_70—78_ and D1R_808—817_ epitopes in rOVA-3 (Figs. 7A and B, columns 1 & 2) but not in response to αGC alone (Fig. 7A, columns 3 & 4). Strikingly however, prime boost vaccination with rOVA-3 alone without any added adjuvant resulted in interferon (IFN)-γ producing T cells in ELISpot assays used to measure peptide-specific responses to B8R_70—78_ and D1R_808—817_ epitopes but not control C4R_70—78_ (Fig. 7B). This response accounted for about a third to one-half of the response to αGC-adjuvanted rOVA-3 (Fig. 7B).

To determine the source of adjuvancy, purified rOVA-3 was subjected to endotoxin removal by using poly(ε-lysine)-coupled cellulose chromatography. This additional step reduced the endotoxin level to ~ 1.0 endotoxin units in the rOVA-3 preparation used in this experiment. This endotoxin level is considered acceptable for in vivo use in mice^102, 103^. Prime boost vaccination with partially detoxified rOVA-3 resulted in B8R_70—78_ and D1R_808—817_ epitopes but not control C4R_70—78_ or K6L peptides. This response required αGC as the adjuvant (Fig. 7C). Hence, endotoxins in the rOVA-3 preparation may have contributed to the molecular signature of CD8^+^ Trm elicited by subunit vaccination described herein.

### Concluding remarks

We conclude that subunit vaccines elicit a robust CD8^+^ Trm response. We reported previously that the resulting Trm response is highly protective^15^. Here we provided evidence that the subunit vaccine also imprints a Trm-lineage specific transcriptome signature. Others showed that this lineage-specific signature was driven by the master transcription factors Runx3 and Hobit^3, 49–51, 66, 69, 70^. Hence, the subunit vaccine described here induces a similar gene regulatory network similar to that induced by natural infections or microbial vaccination regimens. Nonetheless, a few differences were noted between the published Trm transcriptomes and this study. The reason for the differences is unknown but worthy of further investigation. A clear limitation of the current study was the absence of head-to-head comparison between Trm cells induced by subunit vaccination and those elicited by virus infection. Such a side-by-side comparative study will inform ways to harness subunit vaccination-induced lung IST Trm cells for the development of microbe-free vaccines.

The origins of microbe-free subunit vaccine-induced CD8^+^ Trm transcriptome signature warrant additional studies. So also, the contribution of individual adjuvant and their combination in generating a lasting protective immunity by the installation of Trm cells, and how much different adjuvants influence the Trm transcriptome signature require further studies. Notwithstanding the virtues of the subunit vaccine described herein, one limitation of this study is the response elicited by purified rOVA-3 which is in part attributable to co-purifying endotoxins. Nonetheless, as MPLA is an FDA-approved adjuvant^104, 105^, results reported herein are useful for the development of subunit vaccines deliverable via the intranasal route—e.g., FluMist, nasal sprays of quadrivalent, live attenuated influenza viruses, by leveraging advances in delivery platforms and technologies^106–108^.

## Materials and methods

### Mice

B6-*K*^*0*^*D*^*0*^;B*07:02^*tg*^ (B0702^tg^) transgenic mice were previously described^26^. B0702^tg^ mice were bred and maintained in H-2K^b^ and H-2D^b^ deficient background as described previously^26, 29^.

### Ethics and approval

All mouse crosses and experiments complied with the M160174-00, V/17/002, and V1900038-00 protocols approved in accordance with relevant guidelines and regulations stipulated by the Vanderbilt University Institutional Animal Care and Use Committee.

The results of the current study are reported in accordance with ARRIVE guidelines.

### Viruses and infections

Western Reserve (VACV-WR; VR-119, ATCC) and MVA (VR-1566, ATCC) strains of VACV were grown in and titrated with BSC-40 cells. Influenza virus strain A/34/PR/8 (H1N1; PR8, ATCC) was grown in MDCK cells and titered on LLC-MK2 cells.

Ketamine-xylazine anesthetized B0702^tg^ mice were inoculated i.n. with sublethal dose of VACV-WR strain (1 × 10^5^ pfu). For influenza virus infection, anesthetized C57BL/6 mice were inoculated i.n. with 50 pfu or i.p. with 2.2 × 10^5^ PFU A/34/PR/8. Infected mice were monitored daily for morbidity, and those losing over 30% of initial body weight were euthanized per IACUC protocol. Mock infection with PBS served as the negative control. Lungs and spleen from mock and virus infected mice were harvested after 7–10 days post infection (p.i.).

### Subunit vaccines and prime boost vaccinations

The L4R antigen has been described^29^. L4R-b8r DNA was generated by substituting the region within L4R DNA that encodes the FPRSMLSIF epitope with that encoding the FPKNDFVSF (B8R_70–78_) epitope. Recombinantly produced antigens were purified to high purity as described^29,52^.

The design of recombinant OVA (rOVA-3) antigen in which the original cryptic, OT-I and OT-II epitopes were replaced with C4R_70–78_, B8R_70–78_ and D1R_808–817_ epitopes, respectively, was described previously^46^. rOVA-3 was produced and purified as described^29, 46,52^. The resulting protein was refolded by dialysis using the method described previously^15, 29, 52^. Subunit vaccines were prepared fresh for each immunisation. B0702^tg^ mice were primed and boosted by the i.n. route with 70–100 μg rOVA-3 mixed with 1 μg αGC. In some experiments, rOVA-3 was subjected to detoxification by poly(ε-lysine)-coupled cellulose chromatography for high-capacity endotoxin removal according to manufacturer’s instructions (Thermo-Fisher Pearce). Endotoxin levels after detoxification was measured with a Chromogenic Endotoxin Quant Kit -endpoint assay according to manufacturer’s instructions (Pierce). We estimated that detoxification of rOVA-3 by poly(ε-lysine)-coupled cellulose chromatography resulted in ~  ≤ 1 endotoxin units (10 EU = 1 ng endotoxin). It is estimated that a 20 g mouse can tolerate up to 200 ng of endotoxin^103^.

Lungs and spleen from mock and poxvirus challenged mice were harvested after 7–10 days post challenge. Lungs and spleen from subunit vaccination experiments were harvested on days indicated in the experimental strategy of relevant figures.

### Generation of peptide-HLA class I tetramers

Procedures for the production of recombinant human β2 m and B0702, MHC-I refolding with the UV sensitive conditional peptide AARG-J-TLAM (J, 3-amino-3-(2-nitro)phenyl-propionic acid, which is a UV-labile β-amino acid residue), biotinylation and purification of refolded HLA-class I monomers, UV-mediated peptide exchange of the conditional peptide with VACV-derived peptides were performed as described previously^29, 109^. VACV peptide exchanged B0702 (pB0702) monomers were tetramerized with either PE-, APC-, PE-Cy7-, APC-Cy7-strepatavidin (Invitrogen) or BV421-streptavidin (Biolegend) conjugated fluorochromes as described^29, 109^. For dual color encoding, each pB0702 monomers were tetramerized with two fluorochromes. Background staining was determined by irrelevant HMPV-derived CD8^+^ T cell-reactive epitope/B0702 tetramer binding or by staining of mock infected samples.

### Histology and immunohistochemistry

Harvested lungs were fixed in 10% formalin, embedded in paraffin, 10 μm-thick sections cut, and stained with hematoxylin and eosin (H&E). Immunohistochemistry for T cell infiltration of lungs sections was performed on the Leica Bond Max IHC stainer. Slides were deparaffinized. Heat induced antigen retrieval was performed on the Bond Max using their Epitope Retrieval solution for 20 min. Slides were incubated with anti-CD3 antibody (Santa Cruz) for one hour at 1:600 dilution. The Bond Intense R detection system was used for visualization. Stained tissue sections were examined using an Olympus BX41 microscope with Plan Achromat objectives 20X/0.5, 60X/0.90; images were captured with a Spot Flex digital camera using Diagnostic Instruments Spot Advanced acquisition software. Adobe Photoshop was utilized for white balancing and resizing of images.

### Intravital staining and flow cytometry

Intravital staining was performed as described^42, 43^ with the following modification. Mice were injected i.v. with 2 μg of anti-CD45-APC mAb. After 3--5 min, lungs and spleen were harvested and mononuclear cells prepared as described previously^15^.

### Tetramer and antibody staining

All mAbs used for the study are listed in Table S2. Single cell suspensions (2–3 × 10^6^) were incubated with Ghost Voilet 510 viability dye (Tonbo Bioscience) in PBS containing 50 nM Dasatinib (LC laboratories) to differentiate between live/dead cells. After washing cells with FACS buffer (2% v/v FBS and 50 nM Dasatinib in PBS) once, cells were incubated in FACS buffer containing 0.2 μg anti-CD16/CD32 mAb for 15 min on ice to block mAbs from binding to Fc receptors. Cells were then incubated in the dark with fluorochrome-conjugated mAbs to detect surface markers. After 45 min, cells were washed twice with FACS buffer. Surface-stained cells were incubated with 0.25–0.5 μg/ml pB0702 tetramers in PBS containing 50 nM Dasatinib. Flow cytometric data were acquired using FACSCanto II (BD Biosciences) and FACS (.fcs) files were analysed with FlowJo software (Tree Star).

### ELISpot assay

Splenocytes (2 × 10^6^/ml) from prime boost immunized mice^29^ were plated in triplicates and co-cultured with different concentrations of peptides shown in Fig. 3C or with medium alone for 48 h. Interferon (IFN)-γ secreted by antigen stimulated cells were measured and quantified by ELISpot assay as previously described^110, 111^ using 1–1.5 μg/ml anti-mouse IFN-γ mAb capture and detection pairs (see Table S2).

### Cell sorting, RNA isolation, and sequencing

After intravital staining, harvested lungs were digested with collagenase, and live mononuclear cells were prepared using Ficoll density gradient centrifugation to remove dead cells. Briefly, 1–10 × 10^6^ cells in 7 ml of pre-warmed tissue culture medium were carefully underlaid below a 3.5 mL Ficoll-Histopague cushion in a 15 ml Falcon tube. Density gradient was formed by centrifugation 1300 rpm for 30 min at room temperature with no acceleration or brake. Spleens from the same mice were also harvested and mononuclear cells prepared. Cells were pooled from three mouse lungs and spleens. Single cell suspensions were incubated with Ghost Violet 510 viability dye (Tonbo Bioscience) in PBS containing 50 nM dasatinib (LC laboratories) to differentiate live from dead cells. After washing cells with FACS buffer (2% v/v FBS and 50 nM dasatinib in PBS) once, cells were incubated in FACS buffer containing 0.2 μg anti-CD16/CD32 mAb for 15 min on ice to block mAbs from binding to Fc receptors. Next, cells were washed and co-stained with pB0702 tetramers (see Table S1) and anti-CD8α-PerCP Cy5.5, -B220-FITC for 45 min. Cells are washed twice with 2% FBS containing PBS and sorted immediately. The number of cells sorted from each pooled sample are provided in Table S1. Cell sorting was performed using FACS Aria III (BD). Purity of sorted cells was ~ 95%. The gating strategy for FACS analysis is presented in Fig. 3. Detailed information of antibodies used in this study is summarized in Table S5. Whilst each replicate and memory subset were maintained separately, B8R_70—78_ and D1R_808—817_ tetramer-reactive were pooled to have sufficient IST Trm cells for RNA isolation. RNA was isolated according to the manufacturer of NEBNext® Ultra™ II Directional RNA Library Prep Kit (New England BioLabs).

One microgram total RNA from each sample was used for RNAseq. Integrity number of the isolated RNA was 8.3—ranging from 7.9 to 8.8, when assayed on an Agilent 2100 Bioanalyzer. Poly(dA)-positive fraction was purified and randomly fragmented, converted to double stranded cDNA and processed through subsequent enzymatic treatments of end-repair, dA-tailing, and ligation to adapters with NEBNext Ultra II Directional RNA Library Prep Kit for Illumina as recommended by the manufacturer (New England BioLabs). Adapter-ligated library was generated by PCR with Illumina PE primers (New England BioLabs). The resulting purified cDNA libraries were applied to an Illumina flow cell for cluster generation and sequenced on an Illumina instrument by following manufacturer's protocols.

### Bioinformatics

The sequence data quality was checked using FastQC and MultiQC software^112, 113^. The data were checked for base call quality distribution, % bases above Q20, Q30, %GC, and sequencing adapter contamination. All the samples have passed QC threshold (Q30 > 90%). Raw sequence reads were processed to remove adapter sequences and low-quality bases using Trimgalore^114^. The QC-passed reads were mapped onto indexed Mouse reference genome (GRCm38.90) using STAR v2 aligner^115^. The PCR and optical duplicates were marked and removed using Picard tools^116^. Gene level expression values were obtained as read counts using featureCounts software^117^. Expression similarity between biological replicates was checked by Spearman correlation.

Differential expression analysis was carried out using edgeR package after normalizing the data based on trimmed mean of M values^118^. Genes with log_2_ fold change ≥ 1.2–1.5 and adjusted *p*-value ≤ 0.05 were considered significant.

Gene ontology (GO) was applied to identify characteristic biological attributes of RNAseq rwa data. Separate GO Enrichment Analysis using ClusterProfiler R package for up-regulated and down-regulated genes was performed. The output of these analyses was parsed into three categories according to the GO term enrichment analysis for biological processes, cellular components, and molecular functions^119–121^. GO pathway terms with multiple test adjusted *p* value ≤ 0.05 were considered significant.

### Statistics

Data comparisons were performed using Prism version 5.0 (GraphPad software). Where indicated, multiple group comparisons were performed using 1-way ANOVA with Turkey post-test. The descriptive statistics mean ± SEM or mean ± SD was provided for continuous variables as noted. Wilcoxon rank sum test or two-sample t-test was applied to two-group comparisons or the post hoc group comparisons in ANOVA; all tests were two-tailed and unpaired.

## Supplementary Information

Supplementary Information 1.

Supplementary Information 2.

## Data Availability

The datasets generated in the current study are available from NCBI Sequence Read Archive (SRA) under the accession numbers SAMN25818777—SAMN25818788 (see Table S6 for table of contents). Processed data files are provided as supplementary Microsoft Excel files: expression counts (Table S7), normalised expression counts (Table S8), and differential expression (DE) analysis statistics (Table S9).
