# Supplementary material for: A molecular signature of lung-resident CD8+ T cells elicited by subunit vaccination
Source: Sci Rep. 2022 Nov 9;12:19101. doi: 10.1038/s41598-022-21620-7 (PMC9645351; doi:10.1038/s41598-022-21620-7)
Supplement: Supplementary file 1 — Supplementary Information 1. [file 41598_2022_21620_MOESM1_ESM.pdf]

SUPPLEMENTARY MATERIALS: TABLES AND FIGURES

A molecular signature of lung-resident CD8$^+$ T cells elicited by subunit vaccination

Running Title: Subunit vaccine-induced molecular signature of lung CD8$^+$ Trm

Naveenchandra. Suryadevara[1,2], Amrendra Kumar[1,2], Xiang Ye[2], Meredith Rogers[2,4], John V. Williams[2,4,5], John T. Wilson[3], John Karijolich[2] & Sebastian Joyce[1,2,6]

[1]Department of Veterans Affairs, Tennessee Valley Healthcare Center, Nashville, Tennessee 37212, USA; [2]Department of Pathology, Microbiology, & Immunology, Vanderbilt University Medical Center, Nashville, Tennessee 37232, USA; [3]Department of Chemical and Biomolecular Engineering & Department of Biomedical Engineering, Vanderbilt University, Nashville, Tennessee 37212, USA; [4]Department of Paediatrics, University of Pittsburgh School of Medicine, Pittsburgh, Pennsylvania 15224
[5]Institute for Infection, Immunity, and Inflammation in Children (i4Kids), Pittsburgh, Pennsylvania 15224

[6]Corresponding author: Sebastian Joyce < sebastian.joyce@vumc.org >

SUPPLEMENTARY TABLES

TABLE S1. Cell yields post flow sort				
**pB7.2-reactive CD8⁺ T cell**	**Replicate 1** mouse 1—3	**Replicate 2** mouse 4—6	**Replicate 3** mouse 7—9	**Replicate 4** mouse 10—12
		**LUNGS**		
$B8R_{70-78}$ IST Trm	5,482	5,605	2,408	11,057
$D1R_{808-817}$ IST Trm	8,097	6,963	1,246	2,497
$B8R_{70-78}$ MV Tem	53,703	24,458	17,027	51,366
$D1R_{808-817}$ MV Tem	77,087	28,914	6,732	15,780
		**SPLEEN**		
$B8R_{70-78}$ Tcm	108,492	42,327	144,520	124,000
$D1R_{808-817}$ Tcm	81,359	41277	93,282	57,000

Each replicate was made of cells purified from three prime boost vacciants mice. As Trm cell numbers were low, $B8R_{70-78}$ & $D1R_{808-817}$ of each memory type were pooled for RNAseq. Three-to-ten cells cells of each of three memory types and of each replicate were processed as described in Materials & Methods.

Supplementary Tables S2—S4: Gene ontology pathway analyses of pairwise comparison of the three lung CD8$^+$ T cell memory subsets. **Sheet 1**—IST Trm *versus* MV; **Sheet 2**—IST Trm *versus* splenic Tm; **Sheet 3**—MV Tem *versus* splenic Tm. Data are provided as a single Excel File

TABLE S5. List of antibodies used in this study

Antigen	Fluorochrome/tag	Clone	Source	Catalogue number
B220	FITC	RA3-6B2	BD Biosciences	553088
B220	PerCP-Cy5.5	RA3-6B2	BioLegend	103236
B220	APC-Cy7	RA3-6B2	BD Biosciences	552094
CD3ε	APC	145-2C11	BioLegend	100306
CD8α	FITC	53-6.7	BD Biosciences	553031
CD8α	PerCP-Cy5.	53-6.7	BD Biosciences	551162
CD16/CD32	purified	93	BioLegend	101302
CD45.2	APC	104	Tonbo Biosciences	20-0454-U100
Ghost Violet 510	V510	na	Tonbo Biosciences	13-0870-T100
IFN-γ	purified	AN18	BioLegend	517902
IFN-γ	biotinylated	R4-6A2	BioLegend	505704
pB7.2 tetramer	PE	custom	in house	na
pB7.2 tetramer	APC	custom	in house	na

na, not applicable

Table S6: Accession information for RNAseq data deposited at Sequence Read Archive (SRA)*

Accession ID	Sample ID	Sample name**	Organism	Deposit date & time
SAMN25818777	1538-AK-1	IST Trm-1	*Mus musculus*	2022-02-09; 18:01
SAMN25818778	1538-AK-2	IST Trm-2	*Mus musculus*	2022-02-09; 18:01
SAMN25818779	1538-AK-3	IST Trm-3	*Mus musculus*	2022-02-09; 18:01
SAMN25818780	1538-AK-4	IST Trm-4	*Mus musculus*	2022-02-09; 18:01
SAMN25818781	1538-AK-5	MV Tem-1	*Mus musculus*	2022-02-09; 18:01
SAMN25818782	1538-AK-6	MV Tem-2	*Mus musculus*	2022-02-09; 18:01
SAMN25818783	1538-AK-7	MV Tem-3	*Mus musculus*	2022-02-09; 18:01
SAMN25818784	1538-AK-8	MV Tem-4	*Mus musculus*	2022-02-09; 18:01
SAMN25818785	1538-AK-9	Splenic Tcm-1	*Mus musculus*	2022-02-09; 18:01
SAMN25818786	1538-AK-10	Splenic Tcm-2	*Mus musculus*	2022-02-09; 18:01
SAMN25818787	1538-AK-11	Splenic Tcm-3	*Mus musculus*	2022-02-09; 18:01
SAMN25818788	1538-AK-12	Splenic Tcm-4	*Mus musculus*	2022-02-09; 18:01

**SRA will release the deposited RNAseq data immediately upon acceptance of the manuscript*

***Tcm, central memory T cell; Tem, effector memory T cell; Trm, tissue resident memory T cell; MV, marginated vascular; IST, interstitial*

SUPPLEMENTARY FIGURES

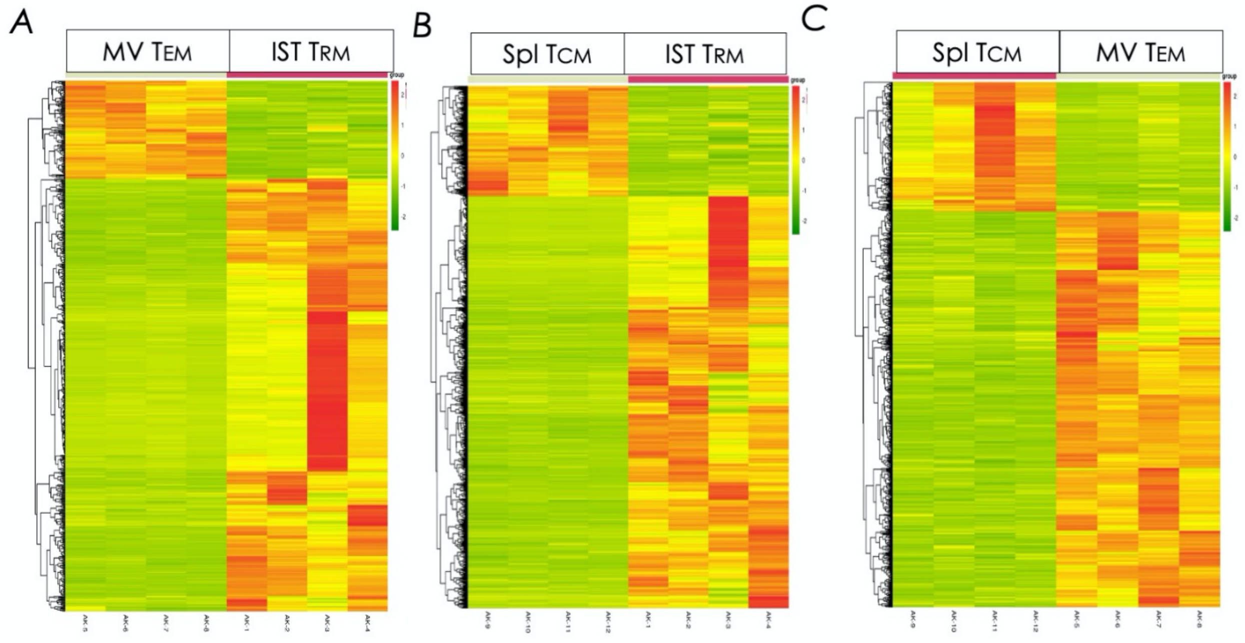

Figure S1: Heatmaps illustrating the relative expression of 17,000 gene transcripts differentially expressed within $CD8^+$ IST Trm, MV Tem, and splenic Tm cell subsets using bulk RNA-seq data. Gene clusters were ordered by K-means clustering analysis. Heatmaps were generated by comparing individual samples from each group MV Tem *vs*. IST Trm (**A**), splenic Tm *vs*. IST Trm (**B**), and splenic Tm *vs*. MV Tem cells. Red, up regulated genes; green, down regulated genes.

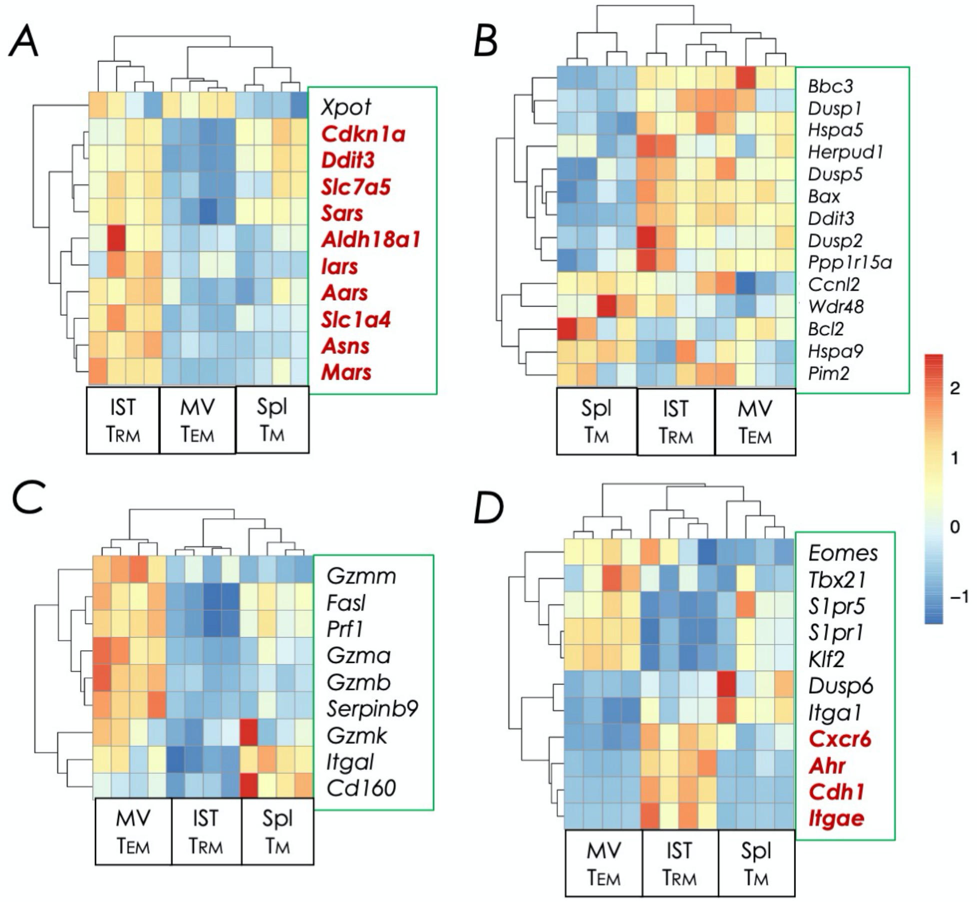

Figure S2: Heatmaps show the expression of selected genes. Expression was normalized as Z score to illustrate differentially expressed genes of individual samples comparing CD8+ IST Trm, MV Tem, and splenic Tm cells. Gene sets shown here were those reported by Hayward et al. (*Nat. Immunol.* ***21****: 309—320, 2020*). Trm-specific genes are indicated in red; red, up-regulated genes; blue, down regulated genes.
